# PETS PROVIDE SIGNIFICANT SUPPORT TO OLDER ADULTS LIVING ALONE: RESULTS FROM THE NATIONAL POLL ON HEALTHY AGING

**DOI:** 10.1093/geroni/igz038.207

**Published:** 2019-11-08

**Authors:** Cathleen M Connell, Erica Solway, Preeti Malani, Dianne Singer, Matthias Kirch, Jeff Kullgren, Mary R Janevic

**Affiliations:** 1 University of Michigan School of Public Health, Ann Arbor, Michigan, United States; 2 Institute for Healthcare Policy and Innovation, University of Michigan, Ann Arbor, Michigan, United States; 3 University of Michigan Medical School, Ann Arbor, Michigan, United States; 4 University of Michigan, Ann Arbor, Michigan, United States

## Abstract

Human-animal interaction has been linked to health and social benefits for older adults. While pets can play a positive role in healthy aging, not all pet owners experience the same benefits. The present study uses data on how pets impact the well-being of older adults, including those living alone from the National Poll on Healthy Aging (NPHA), a nationally representative household survey conducted in October 2018. A randomly selected, stratified sample of adults age 50 to 80 (n=2,051) completed the survey online. Over half reported having a pet; the majority had dogs (70%) and cats (50%). Companionship was the main reason for getting a pet (52%); the majority believed their pets helped them enjoy life, reduce stress, and connect with others. Pet owners living alone were significantly more likely than pet owners living with others to report that their pet helps them cope with physical or emotional symptoms (72% vs. 58%), feel loved (94% vs. 85%), stick to a routine (76% vs. 60%), take their mind off pain (43% vs. 32%), and have a sense of purpose (82% vs. 72%; all p<.05). Respondents living alone were also more likely to report that pet care strains their budget (26% vs. 17%) and that their pet’s needs take priority over their own (22% vs. 14%; p<.05). Given the important role that pets play in the lives of older adults, efforts to support this relationship (e.g., pet-friendly housing policies, low-cost and mobile veterinary clinics, pet walking and pet sitting services) are warranted.

